# The Mechanical and Tribological Properties of Epoxy-Based Composites Filled with Manganese-Containing Waste

**DOI:** 10.3390/ma15041579

**Published:** 2022-02-20

**Authors:** Sebastian Sławski, Anna Woźniak, Patrycja Bazan, Maciej Mrówka

**Affiliations:** 1 Department of Theoretical and Applied Mechanics, Silesian University of Technology, 44-100 Gliwice, Poland; maciej.mrowka@polsl.pl; 2 Materials Research Laboratory, Silesian University of Technology, 44-100 Gliwice, Poland; anna.wozniak@polsl.pl; 3 Faculty of Materials Engineering and Physics, Institute of Materials Engineering, Tadeusz Kosciuszko Cracow University of Technology, 31-864 Cracow, Poland; patrycja.bazan@pk.edu.pl; 4 Biotechnology Center, Silesian University of Technology, 44-100 Gliwice, Poland

**Keywords:** epoxy, composite, casting, manganese residue, sustainability, mechanical properties, abrasion, waste management

## Abstract

Waste from large-scale production processes is a growing environmental problem that can potentially be solved by using this waste as fillers in polymeric composites to improve the mechanical and tribological properties of polymeric matrixes. This paper presents research concerning how the introduction of fillers in the form of manganese residue and manganese(II) oxide changes the mechanical and tribological properties of epoxy composites produced by gravity casting. The research was carried out for composites with 2.5 wt.%, 5 wt.%, and 10 wt.% of fillers. Properties such as the density, hardness, resilience, flexural strength, deflection, flexural modulus, tensile strength, elongation at break, and Young’s modulus were determined. Moreover, based on the ball-on-plate test, the wear volume and friction coefficients of the tested materials were determined. Microscopic images of the abrasion profiles were also obtained. The geometry of the wear paths was measured with a profilometer, and the results showed that introducing fillers reduced the abrasive wear of the composites; however, in all cases, the fillers decreased the strength of the tested materials.

## 1. Introduction

High-tonnage industrial wastes are a growing problem throughout the world because they are often stored in heaps located near cities and have negative impacts on the environment. The manganese ore production worldwide is about six million tons per year (high grade 35% Mn), while manganese alloy production is about eight million tons per year [1]. Currently, about 10–12 t of electrolytic manganese residue is produced for every ton of electrolytic manganese metal, and this level of waste is rising rapidly due to the continued decrease in global rhodochrosite ore-grade quality [2]. The current allowable disposal method for manganese waste is landfill, which can cause human health problems and environmental problems such as soil contamination, river or groundwater pollution, and other serious environmental pollution [3,4]. There are several ways to reuse these industrial wastes, one of which is to use them as fillers in polymeric materials. Chowaniec et al. used industrial wastes as fillers to change the mechanical and tribological properties of a polymeric matrix [5]. Depending on the matrix, the filler and its amount in a matrix gave the resulting material various new properties. The application of various materials as a matrix or reinforcement gives countless possibilities for modifying the properties of composites [6]. Over the last few years, there has been a significant increase in the use of composite materials. Polymeric composite materials have been increasingly used as construction materials because of their strength, relatively low weight, and ease of forming various shapes [7]. Moreover, composites show resistance to aging, i.e., the influence of the environment, which allows them to be used in places where steel and aluminum alloys cannot [8,9]. As previous research has shown [10,11], the introduction of industrial waste into silicone-based composites decreases their mechanical properties, but improves their tribological properties. The authors decided to assess the influence of industrial waste on the mechanical and tribological properties of epoxy-based composites, which are widely used in many different industries, such as aviation [12,13], energy (e.g., wind turbines) [14], railways [15], marine applications [16], and as protection [17,18,19]. Among the main advantages of epoxy resins is their remarkable ability to adapt to a wide range of applications, from simple functional surface coatings to building structural elements [20].

Research concerning the abrasive wear of epoxy-based composites filled with multi-walled carbon nanotubes (MWCNTs) showed that the weight loss was much lower in the case of the filled composite compared with the neat epoxy resin [21]. Moreover, epoxy-based composites filled with MWCNTs (up to 2.0 wt.%) have a higher tensile strength compared with the neat epoxy resin [22]. Krzyzak et al. investigated changes in epoxy composites’ mechanical and tribological properties when filled with Al_2_O_3_ grains of various sizes. The greatest reduction in the abrasive wear of the tested samples was noticed in the case of the samples filled with alundum with grain sizes of F220 and F240. The addition of a physical friction modifier influenced some of the composite’s mechanical properties. It was noticed that the Young’s modulus increased with the filler content and upon decreasing the grain size. It was also observed that the highest values of the max principal stress for the tested samples in most cases were obtained for 10% alundum (the max principal stresses decreased upon further increasing the filler content) [23]. Szczepaniak et al. investigated the mechanical properties of an ablative composite on an epoxy matrix base with aerogel particles. As a result of adding the aerogel, the structural and mechanical properties of the composite changed, and its strength was significantly reduced [24]. Stabik et al. noticed that the introduction of coal particles into an epoxy resin slightly decreased the abrasion resistance compared with the epoxy resin without fillers [25]. Yemam et al. noticed that the use of sand washing waste as fillers (up to 20 wt.%) in an epoxy mortar improved its compressive strength, flexural strength, and modulus of elasticity [26]. Bagherzadeh et al. investigated epoxy-based composites filled with SiO_2_ and CaCO_3_ particles. They noticed that for both nanoparticles, the properties were higher at lower contents. As the content rose, the adhesion and mechanical properties decreased due to nanoparticle agglomeration [27]. Mahadeva Raju et al. noticed that the tensile and compressive strengths were improved in epoxy-based composites upon increasing the fly ash content [28]. Hodul et al. investigated the mechanical and tribological properties of epoxy-based composites filled with various fillers such as quartz sand, waste glass from solar panels, waste from the production of mineral insulation boards, fly ash, neutralization sludge, and waste foundry sand. They noticed that the type of filler had a more significant influence on the resulting properties of the mixture than the type of resin used. They also stated that fillers with coarser grains with a more uniform geometric shape had a positive influence, especially on the compressive strength due to less compression of the filler grains in the epoxy composite. Alternatively, epoxy composites filled by finer fillers showed a better flexural strength, abrasion resistance, and impact resistance [29]. To summarize, the introduction of physical fillers into an epoxy matrix can improve the mechanical and tribological properties of the resulting materials. The degree of improvement will affect the future applications of composite materials. In some applications, the dynamic behavior of filled composite will be different [6,30], and this may exclude the possibility of using newly created materials.

In this article, we assessed the influence of the introduction of high-tonnage industrial wastes on the mechanical and tribological properties of epoxy-based composites. Polymeric materials used in sliding motion joints play an essential role in technological development [31], so it is important to investigate the tribological properties of newly created composite materials. Fillers in the form of manganese(II) oxide (MnO) and manganese residue (MR) were obtained from the Zakłady Górniczo-Hutnicze “Bolesław” S.A. Capital Group (Bukowno, Poland). These materials are waste products of the electrolysis process when used with steel–lead alloy anodes. The use of waste materials obtained from high-tonnage industrial processes is an important part of sustainability and caring for the environment. Currently, paths for the recycling of wastes in various branches of the economy are being sought to reduce the need to store and process industrial waste. The physicochemical properties of the manufactured composites filled with MnO and MR fillers were assessed. In the literature, there is a lack of research concerning the reuse of MnO and MR waste as a filler in epoxy-based composites. The performed research brings information about the influence on the change in mechanical and tribological properties of epoxy-based composites. This can contribute to the start of the reuse of MnO and MR waste as a filler in polymer composites, which will have a positive impact on the environment.

## 2. Materials and Methods

### 2.1. Materials

Epoxy resin LG285 [32] was used as the matrix. The resin was mixed with by a manufacturer-dedicated HG285 hardener in a 100:40 weight ratio. A set of samples was made for both the reference material (neat epoxy resin with hardener—REF) and for the resin with added fillers in the form of MR and MnO. The fillers were production waste and were supplied by Zakłady Górniczo-Hutnicze “Bolesław” S.A. Capital Group (Bukowno, Po-land). The MnO filler consisted of 92.5% grains with a diameter below 0.1 mm and 7.5% grains with a diameter in the range of 0.1–0.315 mm. The MR filler consisted of grains with a diameter below 0.1 mm (100%). The chemical composition of the MR and MnO fillers are shown in Table 1 and Table 2, respectively.

Different amounts of fillers (2.5 wt.%, 5 wt.%, and 10 wt.%) were added to the epoxy matrix. The sample designations depended on the composition and are listed in Table 3.

The materials were produced using the gravity casting method. The mixtures were poured into silicone molds, which were the negative of the samples used in the research. In the case of the composite materials, to minimize sedimentation, the fillers were added 20 min after mixing the resin with the hardener. Cast composites were left in the silicone molds for 24 h. After the samples were removed from the molds, they were crosslinked in a laboratory dryer SLW53STD (POL-EKO-APARATURA, Wodzisław Śląski, Poland) at 80 °C for 2 h. Then, the samples were subjected to a machining process to compensate for the unevenness created during the production process.

### 2.2. Methods

**Figure 1:**
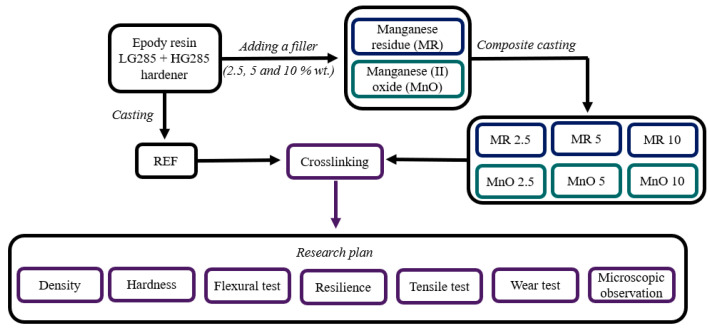
Research methodology scheme.

The aim of the research was to investigate the effect of introducing MR and MnO fillers on the mechanical and tribological properties of the newly created composite materials compared with the reference material (neat epoxy matrix). The mechanical properties of the manufactured materials were determined based on the results of two tests, such as density testing by hydrostatic weighing, hardness, resilience, three-point flexural tests, and tensile tests. The comparison of the tribological properties was made based on the ball-on-plate test. The scheme of the developed research methodology is presented in Figure 1.

The hydrostatic weighing method was used to determine the density of the tested samples. During the test, an analytical balance with a density measurement kit Adventure Pro AV264CM (OHAUS Europe GmbH, Nänikon, Switzerland) was used. The study was conducted in accordance with EN ISO 1183-1 [33]. Five samples from each composition were used. Each sample was weighed twice. The first measurement was carried out with a sample placed on a pan in the air (*m*_1_). The second measurement was carried out for a sample immersed in a liquid of a known density (*m*_2_). During the tests, water was used as the liquid with a known density ($\rho_{H_{2}O}=0.997$ g/cm^3^). The densities of the newly created composites (ρ) were determined using Formula (1):

(1) $$\rho =\rho_{H_{2}O}\cdot \frac{m_{1}}{\left(m_{1}-m_{2}\right)},$$

The Shore D hardness test was carried out in accordance with EN ISO 868 [34]. The hardness durometer Shore D TI-D (SAUTER GmbH, Wutöschingen, Germany) was used during the test. Five measurements were made for each composite, maintaining a distance of at least 10 mm from the sample edge.

The resilience of the tested materials was determined by the Charpy test, which was carried out using an Impact Hammer BPI-25COMC.10 (Zwick/Roell, Ulm, Germany) in accordance with EN ISO 179-1 [35]. The test samples were not notched. The test was performed with a support spacing of 62 mm. The impact strength was measured during edge impact for five specimens from each composition. The test was carried out using a hammer with a nominal impact energy of 1 J.

The flexural properties were tested by a three-point flexural test in accordance with EN ISO 178 [36]. A universal testing machine Autograph AGS-X (Shimadzu, Kyoto, Japan), with a measuring range of up to 10 kN, was used during the test. The test speed was set to 5 mm/min. The test was carried out with a support spacing of 64 mm.

Tensile tests (in accordance with EN ISO 527-2 [37]) were carried out with an MTS Criterion Model 43 universal testing machine (MTS System Corp., Eden Prairie, MN, USA), with a measuring range of up to 30 kN using the MTS axial extensometer. The test speed was set to 2 mm/min. During the research, samples of the 1A type were used [37].

The analysis of the MR and MnO powders was performed using scanning electron microscopy Supra 35 (Zeiss, Oberkochen, Germany), equipped with a secondary electron (SE) detector. The observations were carried out at a magnification of 1000–5000× and an accelerating voltage of 10 kV. Additionally, microchemical analysis by the point-by-point method was performed using energy-dispersive spectroscopy EDS (EdaX, Mahwah, NJ, USA).

The surface topography was analyzed using digital 3D microscopy Leica DVM6 (Leica Microsystem, Wetzlar, Germany).

The wear tests were conducted using a CSM tribometer (CSM Instruments, Switzerland) by the ball-on-plate method. A stainless steel ball with a diameter of 5.6 mm was used as a counter specimen. The wear test was carried out using a normal load Fn = 20 N under a frequency of 1.86 Hz. The measured distance was 35 m. Based on the performed measurements, the value of the coefficient of friction µ was determined. The wear resistance of all tested samples was evaluated based on the cumulative wear volume V (mm^3^) and specific wear rate W ((mm^3^)/(N min)). The wear volume V was determined from the cross-sectional area of the wear tracks, which were measured using a profilometer Surtronic 35 (Taylor Hobson, Leicester, United Kingdom). The specific wear rate was calculated using Formula (2), where *V* is the wear volume (mm^3^), *F_n_* is the force (N), and *S* is the total sliding distance (m):

(2) $$W=\frac{V}{F_{n}\cdot S},$$

Images of the wear tracks were taken using a 3D digital microscope (Leica DVM6). Additionally, the volume of the wear tracks was measured using a profilometer Surtronic 35 (Taylor Hobson, Leicester, UK).

## 3. Results

**Figure 2:**
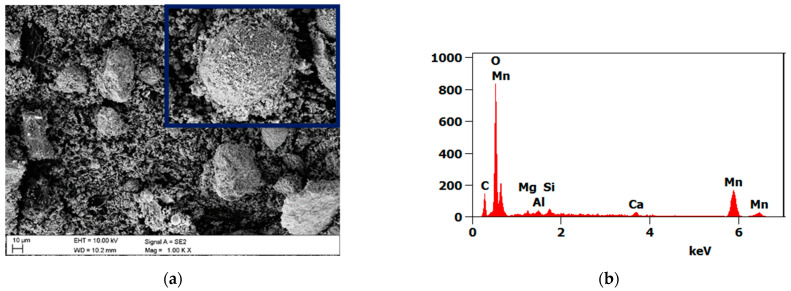

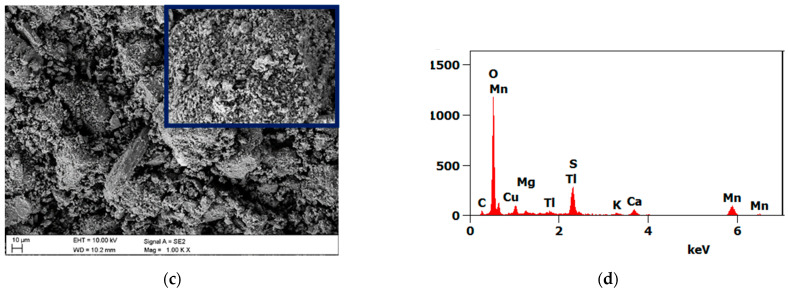
Powder observations and EDS analysis of (**a**,**b**) MR and (**c**,**d**) MnO.

The results of the microscopic analysis of the powders used as a filler are presented in Figure 2. Both the MR and MnO powders were irregular in shape and size (Figure 2a,c). The microchemical analysis showed a high concentration of Si, Mg, and Al in the MR powder (Figure 2b) and a high concentration of Ti in the MnO powder (Figure 2d). The MnO powder was characterized by larger particles than the MR powder.

**Figure 3:**
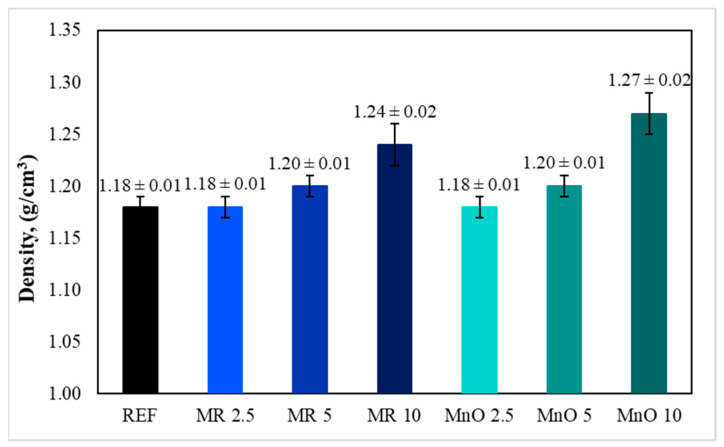
Results of density measurements.

Based on Formula (1), the density of the tested materials was determined, and the results are presented in Figure 3, which are the average of the measurements performed on five samples for each composition.

The average measured density of the reference material was 1.18 g/cm^3^. The density of the material did not change when 2.5 wt.% of fillers were added. Compositions containing 5 wt.% and 10 wt.% of fillers had a higher density. For compositions containing 5 wt.% of fillers, the average density was 1.20 g/cm^3^. The highest density (1.27 g/cm^3^) among the tested materials was observed for the composition containing 10 wt.% of MnO filler. The density of the composition containing 10 wt.% of filler in the form of MR was 1.24 g/cm^3^. The obtained results showed that the introduction of the fillers in the form of MR and MnO to the epoxy matrix increased the material’s density. This was because the density of the fillers was greater than the density of the epoxy resin matrix. The obtained results also indicated that the introduction of the MnO filler caused a greater increase in density than the MR filler. The increase of epoxy-based composites’ density after the introduction of physical fillers has also been reported by other researchers [23].

**Figure 4:**
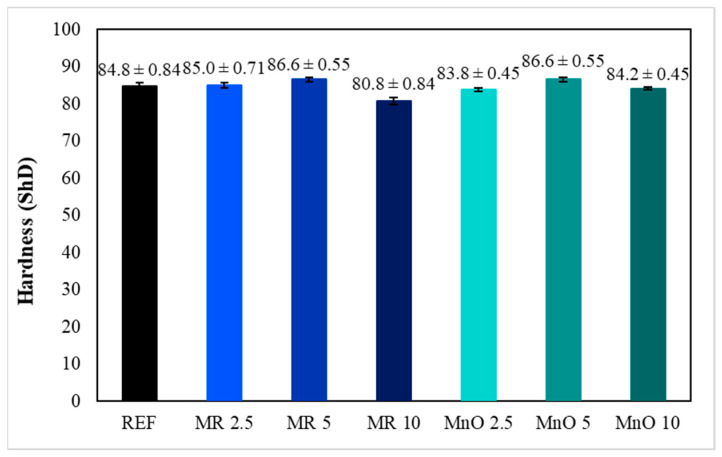
Results of hardness measurements.

The measured hardness values of the tested compositions are presented in Figure 4, which are the average of five measurements for each composition.

The hardness of the reference material was 84.8 ShD. The introduction of 2.5 wt.% of filler in the form of MR increased the material’s hardness by about 0.2 ShD compared with the neat epoxy resin (REF). The introduction of 2.5 wt.% of filler in the form of MnO decreased the material’s hardness by about 1 ShD compared with the neat epoxy resin (REF). The highest increase in material hardness was noticed for 5 wt.% of fillers. In both cases (MR5 and MnO 5), the hardness was 86.6 ShD. For both fillers, adding 5 wt.% of filler was the breaking point. For both fillers, the hardness of the compositions containing 10 wt.% of filler decreased compared with the reference material. The lowest hardness was observed for the MR 10 composition (80.8 ShD). Similar results were obtained by Krzyzak et al. Independent of the Al_2_O_3_ grain size, the hardness of the epoxy-based composites increased slightly when the amount of introduced Al_2_O_3_ filler increased [23]. However, the hardness of epoxy-based composites after the introduction of physical fillers depends on the introduced filler, which was well presented by Horus et al. [29].

**Figure 5:**
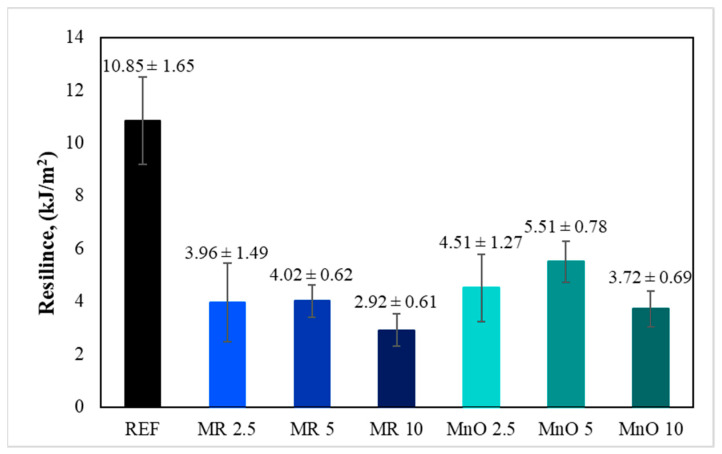
Results of resilience measurements.

The resilience of the tested materials is presented in Figure 5, which was the average of five measurements for each composition.

The resilience of the reference material was 10.85 kJ/m^2^. The introduction of the fillers in the form of MR and MnO into the epoxy matrix caused a large decrease in the resilience of the tested compositions. The resilience of the filled compositions was 2–3-times lower than that of the reference material. The resilience of the compositions containing the filler in the form of MR was lower than that of the composition filled with MnO in all cases. The analysis of the results for the compositions containing fillers showed that the break point in both cases was with 5 wt.% of filler. The resilience of the MnO 5 composition was higher than that of the MR 5 composition by about 1.49 kJ/m^2^. The lowest resilience was observed for the composition MR 10 (2.92 kJ/m^2^). It was suspected that the decrease in the resilience of the filled compositions was related to the discontinuity of the matrix caused by the fillers.

Three-point flexural tests were used to determine properties such as the flexural strength, deflection, and flexural modulus. The average values of these properties measured for five samples from each composition are presented in Table 4.

The flexural strength of the reference material was 113.7 MPa. The results showed that the introduction of 2.5 wt.% and 5 wt.% of fillers increased the flexural strength of the composites. The highest flexural strength for both fillers was observed when they were included in the epoxy matrix in the amount of 2.5 wt.%. The flexural strength of the MR 2.5 composition (117.6 MPa) was not much lower than the flexural strength of the MnO 2.5 composition (119.1 MPa). The flexural strength of the compositions containing 10 wt.% of fillers was lower than that of the reference material. The flexural strength of the composition MR 10 (102.8 MPa) was lower than that of the composition MnO 10 (105.1 MPa). The filler content in the epoxy matrix had a similar effect on the results obtained in both cases. However, greater flexural strength was observed for the compositions filled with MnO.

The deflection of the reference material was 6.8 mm, which was the highest value recorded. Introducing fillers into the composition decreased the deflection in all analyzed cases. The deflection of the compositions filled with MR was higher than that of the composition filled with MnO in all analyzed cases. The smallest deflection was observed for composition MnO 10 (4.5 mm). The deflection of composition MR 10 was slightly higher (4.6 mm). The deflection of composition MR 2.5 (6.6 mm) was slightly lower than that of the reference material (6.8 mm). The deflection of composition MR 2.5 (6.6 mm) was the largest among the compositions containing only one filler.

The flexural modulus of the reference material was 3126.9 MPa. A similar tendency was observed for the compositions containing the fillers—the greater the filler content, the greater the flexural modulus was. Higher flexural modulus values were observed in the case of compositions containing the MnO filler. The flexural modulus of the compositions filled with MnO was higher than that of the reference material in all analyzed cases. The highest flexural modulus among the tested materials was observed for MnO 10 (3451.4 MPa). The flexural modulus of MR 2.5 (2964.2 MPa) was smaller than that of the reference material (3126.9 MPa). The flexural modulus of MR 2.5 was the smallest among the tested materials. The highest flexural modulus among the compositions filled with MR was observed for MR 10 (3261.5 MPa).

The compositions containing the MnO filler were characterized by a higher flexural strength and flexural modulus than those filled with MR. In most cases, these values were greater than those of the reference material. The highest flexural strengths were observed in compositions containing 2.5 wt.% of fillers. A decrease in flexural strength below that of the reference material was observed only for the compositions containing 10 wt.% of fillers.

For all tested compositions containing fillers, a relationship between the flexural modulus, flexural strength, and deflection was observed. Regardless of the filler, increasing its wt.% in the composition increased the flexural modulus and decreased the flexural strength and deflection.

The tensile strength, elongation at break, and Young’s modulus of the composites were determined. The average values of these properties measured for five samples from each composition are presented in Table 5.

Introducing fillers into the epoxy resin significantly reduced the tensile strength. The tensile strength of the reference material was 80.5 MPa, which was the highest value among the tested materials. In the case of compositions containing fillers, an approximately two-fold decrease in the tensile strength was observed in all analyzed cases. Upon increasing the filler content, the tensile strength decreased. The highest tensile strength among the compositions containing one of the fillers was observed for MnO 2.5 (46.8 MPa). The tensile strength of MR 2.5 was 43 MPa. For both fillers, the lowest tensile strength was observed when using 10 wt.% of fillers. The tensile strength of MnO 10 was 33.2 MPa, which was the lowest value among the tested materials. Regardless of the wt.% of the fillers, a higher tensile strength was observed when MnO was used as the filler compared to MR. As in the case of flexural strength, the highest tensile strength among the compositions containing fillers was observed when 2.5 wt.% of fillers was added.

The elongation at break of the reference material was 6.7%, which was the highest value among the tested materials. In the case of compositions containing fillers, a more than two-fold decrease in the elongation at break was observed in all analyzed cases. The highest elongation at break in compositions containing fillers was observed for MnO 2.5 (3.4%), and the elongation at break of MR 2.5 was 3.1 %. In other cases, a smaller decrease in the elongation at break occurred in the compositions containing the MR filler. For both fillers, the lowest elongation at break was observed for compositions containing 10 wt.% of fillers. The smallest elongation at break among the tested materials was observed for MnO 10 (2.3 %). Upon increasing the amount of filler, the elongation at break of the tested materials decreased.

The Young’s modulus of the reference material was 3611.8 MPa, which was the smallest value among the tested materials. As the amount of the MR filler increased, the Young’s modulus decreased. When using the MnO filler, the opposite trend was observed. The highest Young’s modulus among the compositions filled with MR was noticed for MR 2.5 (3971.2 MPa). The highest Young’s modulus among the compositions filled with MnO was noticed for MnO 10 (4756.4 MPa). The Young’s modulus of MnO 10 was the highest among the tested materials, and the smallest Young’s modulus was noticed for MnO 2.5 (3714.3 MPa).

The decrease in the composites’ strength (tensile strength, flexural strength, resilience) could be related to the non-uniform dispersion of the filler, which could cause an earlier failure in the matrix under load. The agglomeration of the fillers used and the created discontinuities of the matrix also reduced the material strength, especially at higher filler content. The decrease in the composite strength could be also related to the size of the introduced filler. In the case of nanofillers such as MWCNTs, their introduction into an epoxy matrix causes an increase both in flexural and tensile strength [22,38]. The increase of the flexural modulus and Young’s modulus of the tested composites could be related to the fillers used, the modulus of elasticity of which was greater than the neat epoxy matrix modulus of elasticity.

**Figure 6:**
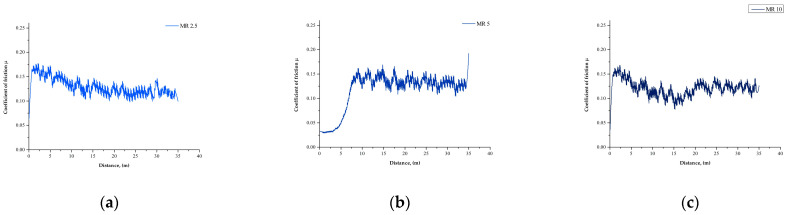

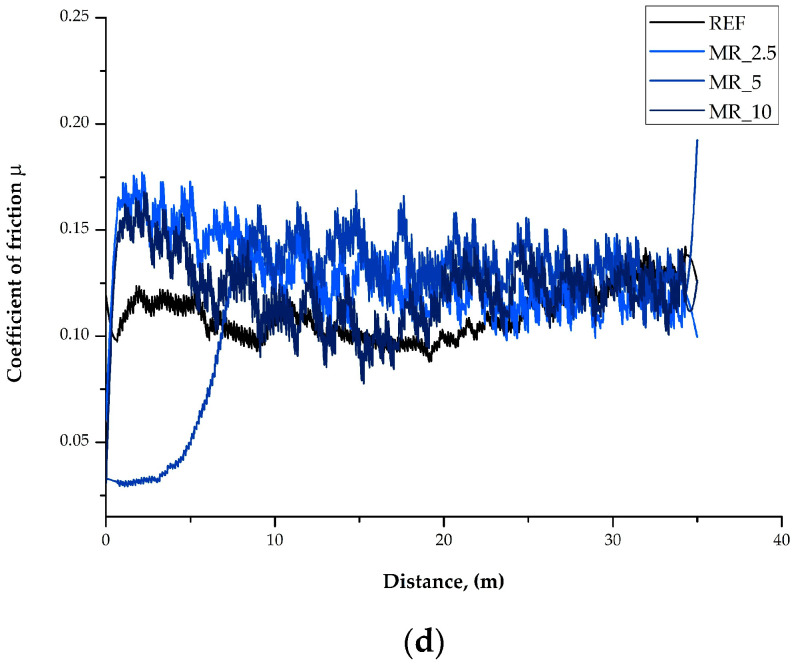
Friction coefficient evolution of the MR samples’ group: (**a**) MR 2.5, (**b**) MR 5, (**c**) MR 10, and (**d**) all samples with the reference sample.

**Figure 7:**
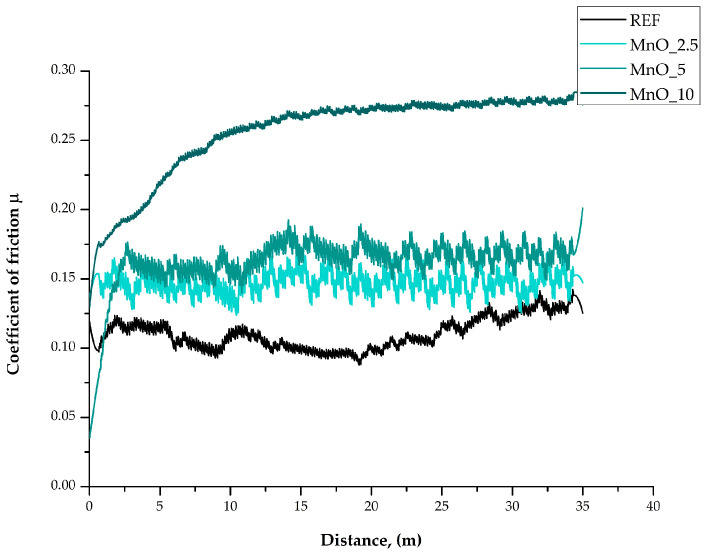
Friction coefficient evolution of the MnO samples’ group.

The coefficient of friction diagrams from the ball-on-plate tests as a function of distance are shown in Figure 6 and Figure 7. For all samples of the MR series, the coefficient of friction rapidly increased in the first stage of the wear resistance test. Next, a decrease in the value was observed. This was associated with the rough surface of the samples and the counter sample (stainless steel ball), which remained in friction contact. Due to the effect of the initial point contact of the friction pair, a high compressive pressure was generated, which resulted in sequential deformation and fragmentation of the surface of the tested samples. This led to faster wear and is a typical sliding wear mechanism. Next, for the samples with 2.5 wt.% and 10 wt.% of MR, a transition was observed: after the minimum coefficient of friction was reached, it began to increase. This could be attributed to the Hertzian phenomenon. For the MR 2.5 and MR 10 samples, the steady-state coefficient of friction (COF) was reached after a distance d > 20 m, while for samples with 5 wt.% of MR filler, the steady-state was reached at a three-times smaller distance d > 7 m. For all samples with MnO fillers (except the MR 5 sample), an increase in the coefficient of friction followed by a decrease and stabilization was observed. For the MnO 5 sample, the steady-state COF was reached after a distance d > 2.5 m and for the MnO 10 sample, after a distance d > 7.5 m. Additionally, for the MnO 2.5 sample, the diagram of the coefficient of friction changed over time and then stabilized.

For all samples with MR as the filler, regardless of the filler concentration, the average coefficient of friction was µ = 0.12, which was similar to the value of the reference material (REF) µ = 0.11. However, for the MnO group, regardless of the filler concentration, a higher coefficient of friction was observed. It should be noted, however, that a significant change occurred only for the samples with 10 wt.% of MnO, and the value was around µ = 0.24.

The characteristic wear resistance is given in Table 6.

The lowest wear resistance parameters were obtained for the reference sample (REF). The mean wear volume V_w_ and the wear coefficient K were 0.1 × 10^−2^ mm^3^ and 1.6 × 10^−4^ mm^3^/N·min, respectively. The use of MR and MnO as the fillers provided wear resistance parameters that were an order of magnitude higher than those of the reference sample. For the samples with MR as the filler, regardless of the concentration, similar values were obtained. The V_w_ and K values of all samples in the MR series were in the range of 3.04 × 10^−3^ to 3.72 × 10^−3^ mm^3^ and from 4.32 × 10^−5^ to 5.31 × 10^−5^ mm^3^/N·min, respectively. However, for the MnO series, the smallest values of V_w_ and K were recorded. Additionally, upon increasing the MnO concentration, the volume and wear coefficient also increased, and for the MnO 10 group, V_w_ = 1.34 × 10^−3^ mm^3^ and K = 1.91 × 10^−5^ mm^3^/ N·min. The increase of epoxy-based composites’ wear resistance after introducing physical fillers has also been reported by other researchers [21,22]. However, in some cases, such as epoxy–hard coal composites, a slight decrease in abrasion resistance with increasing filler content can be noticed [25].

**Figure 8:**
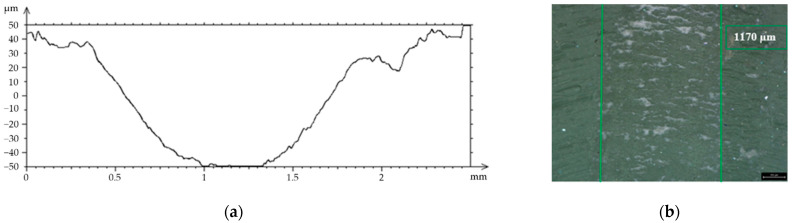
Wear test results of reference samples: (**a**) profile of the wear track; (**b**) surface morphology after the wear test.

**Figure 9:**
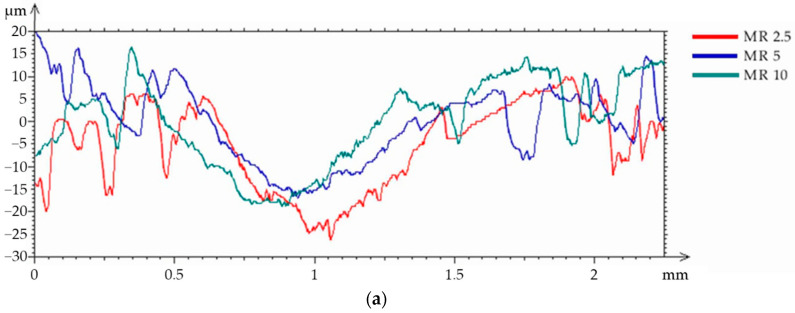

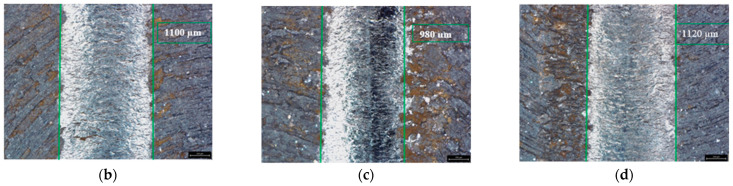
Wear test results of samples with the MR filler: (**a**) profiles of wear tracks and surface morphology of: (**b**) MR 2.5 wear track; (**c**) MR 5 wear track; (**d**) MR 10 wear track.

**Figure 10:**
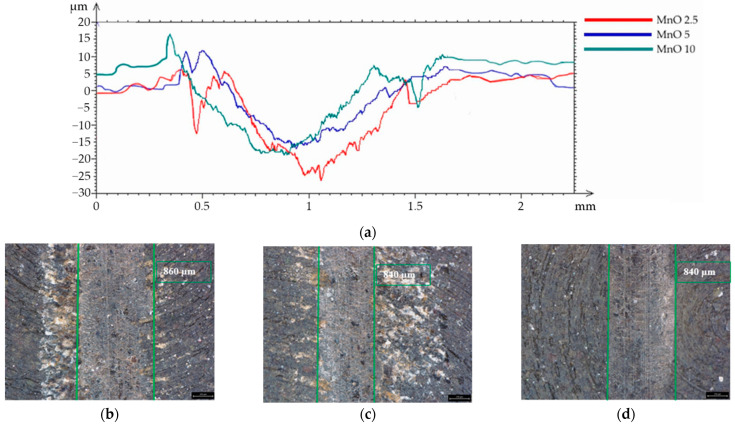
Wear test results of samples with the MnO filler: (**a**) profiles of wear tracks and the surface morphology of: (**b**) MnO 2.5 wear track; (**c**) MnO 5 wear track; (**d**) MnO 10 wear track.

Based on the analysis of the surface topography of the REF after the wear test (Figure 8), it can be concluded that the wear track was regular, and its width was approximately 1170 µm. The analysis of the wear tracks for the samples with MR as the filler showed an increase in the wear debris (Figure 9a–d). The mean width of the wear tracks for the MR 2.5 and MR 10 samples was similar and was approximately 1100 µm. This was similar to the wear track for the references samples. For the MR 5 group, the lowest wear track width was obtained (980 µm), which may indicate lower material wear due to lower pressure between the friction pair, which was also confirmed by the lowest values of the abrasion parameters for the MR series. Based on the analysis of the wear tracks for samples with the MnO filler (Figure 10a–d), the wear track width of all samples was similar and was approximately 860 µm. No increase in the amount of wear debris on the surface was observed. Based on the conducted analyses and observations, it can be concluded that the MnO series samples displayed the optimal frictional wear resistance.

## 4. Conclusions

This research showed that the introduction of MR and MnO fillers to an epoxy resin decreased the wear volume in all analyzed cases. A greater reduction in wear volume was observed for samples containing MnO. The wear paths of compositions containing the MnO filler were narrower than those containing MR filler. However, the introduction of fillers into the epoxy resin was also associated with a decrease in the mechanical properties of the tested compositions, in particular the tensile strength and resilience. The compositions containing the MnO filler were characterized by better mechanical properties than those containing the MR filler. The conducted research showed that the investigated wastes can be used as fillers to reduce the abrasive wear of epoxy resins. However, attention should be paid to changes in the mechanical properties. Better mechanical and tribological properties were observed in the case of using the MnO filler, and the best results were obtained for compositions containing 2.5 wt.% and 5 wt.% of MnO filler. Moreover, the use of fillers in the form of waste from large-scale production processes has a positive effect on the environment.

## Figures and Tables

**Table 1:** Chemical composition of the MR filler.

Compound	MnO_2_	SiO_2_	MnO	ZnSO_4_	Other
Content (%)	72	11	7	5.5	4.5

**Table 2:** Chemical composition the MnO filler.

Compound	MnO	Fe	CaO	Al_2_O_3_	MnO_2_	Other
Content (%)	78	5.9	2.7	1.5	1.3	10.6

**Table 3:** Composite compositions.

Matrix	Filler	Filler Content (%)	Designation
Epoxy resin LG285 + HG285 hardener	-	-	REF
	manganese residue	2.5	MR 2.5
	manganese residue	5	MR 5
	manganese residue	10	MR 10
	manganese(II) oxide	2.5	MnO 2.5
	manganese(II) oxide	5	MnO 5
	manganese(II) oxide	10	MnO 10

**Table 4:** Flexural properties of the tested materials.

Material	Flexural Strength (MPa)	Deflection (mm)	Flexural Modulus (MPa)
REF	113.7 ± 12.4	6.8 ± 1.4	3126.9 ± 159.8
MR 2.5	117.6 ± 4.9	6.6 ± 1.1	2964.2 ± 167
MR 5	117.0 ± 5.7	6.1 ± 0.9	3143.6 ± 67.9
MR 10	102.8 ± 10.6	4.6 ± 0.7	3261.5 ± 217.2
MnO 2.5	119.1 ± 8.2	5.8 ± 0.7	3133.4 ± 145.1
MnO 5	116.8 ± 9.9	5.9 ± 1	3250.7 ± 254.1
MnO 10	105.1 ± 11.6	4.5 ± 0.9	3451.4 ± 105.1

**Table 5:** Tensile properties of the tested materials.

Material	Tensile Strength (MPa)	Elongation at Break (%)	Young’s Modulus (MPa)
REF	80.5 ± 1.4	6.7 ± 0.5	3611.8 ± 88.8
MR 2.5	43.0 ± 9.7	3.1 ± 1.1	3971.2 ± 511.3
MR 5	43.1 ± 4.7	3.2 ± 0.6	3970.2 ± 496.6
MR 10	36.4 ± 3.5	2.8 ± 0.1	3827.1 ± 329.2
MnO 2.5	46.8 ± 2.7	3.4 ± 0.5	3714.3 ± 480
MnO 5	42.6 ± 2.9	2.8 ± 0.2	4186.7 ± 198.6
MnO 10	33.2 ± 7.1	2.3 ± 0.5	4756.4 ± 594.4

**Table 6:** Results of the wear test.

Material	Coefficient Friction µ	Wear Volume V_w_ (mm^3^)	Wear Coefficient K (mm^3^/N min)
REF	0.11	0.11 × 10^−2^	1.6 × 10^−4^
MR 2.5	0.12	3.72 × 10^−3^	5.31 × 10^−5^
MR 5	0.13	3.04 × 10^−3^	4.32 × 10^−5^
MR 10	0.12	3.57 × 10^−3^	5.10 × 10^−5^
MnO 2.5	0.14	1.27 × 10^−3^	1.81 × 10^−5^
MnO 5	0.17	1.26 × 10^−3^	1.80 × 10^−5^
MnO 10	0.25	1.34 × 10^−3^	1.91 × 10^−5^

## Data Availability

The data presented in this study are available upon request from the corresponding author.
